# The Transcription Factor T-Bet Is Required for Optimal Type I Follicular Helper T Cell Maintenance During Acute Viral Infection

**DOI:** 10.3389/fimmu.2019.00606

**Published:** 2019-03-29

**Authors:** Pengcheng Wang, Youping Wang, Luoyingzi Xie, Minglu Xiao, Jialin Wu, Lifan Xu, Qiang Bai, Yaxing Hao, Qizhao Huang, Xiangyu Chen, Ran He, Baohua Li, Sen Yang, Yaokai Chen, Yuzhang Wu, Lilin Ye

**Affiliations:** ^1^Institute of Immunology, PLA, Third Military Medical University, Chongqing, China; ^2^National Clinical Research Center of Kidney Diseases, Jinling Hospital, Nanjing, China; ^3^Cancer Center, The General Hospital of Western Theater Command, Chengdu, China; ^4^Chongqing Public Health Medical Center, Chongqing, China

**Keywords:** T-bet, follicular helper T cells, type I immune response, humoral response, T cell differentiation, transcriptional regulation

## Abstract

Follicular helper T cells (TFH cells), known as the primary “helpers” of the germinal center (GC) reaction, promote the humoral immune response to defend against various pathogens. Under conditions of infection by different types of pathogens, many shared transcription factors (TFs), such as Bcl-6, TCF-1, and Maf, are selectively enriched in pathogen-specific TFH cells, orchestrating TFH cell differentiation and function. In addition, TFH cells also coexpress environmentally associated TFs as their conventional T cell counterparts (such as T-bet, GATA-3, or ROR-γt, which are expressed in Th1, Th2, or Th17 cells, respectively). These features likely indicate both the lineage-specificity and environmental adaption of the TFH cell responses. However, the extent to which the TFH cell response relies on these environmentally specific TFs is not completely understood. Here, we found that T-bet was specifically expressed in Type I TFH cells but not Type II TFH cells. While dispensable for the early fate commitment of TFH cells, T-bet was essential for the maintenance of differentiated TFH cells, promoting their proliferation, and inhibiting their apoptosis during acute viral infection. Microarray analysis showed both similarities and differences in transcriptome dependency on T-bet in TFH and TH1 cells, suggesting the distinctive role of T-bet in TFH cells. Collectively, our findings reveal an important and specific supporting role for T-bet in type I TFH cell response, which can help us gain a deeper understanding of TFH cell subsets.

## Introduction

Because of the complexity and diversity of pathogens, organisms have developed highly organized and well-adapted immune systems to eliminate invaders. To defend against different microorganisms, the immune system elicits optimal responses according to the species of invader (1). For example, intracellular microbes induce type I immune response, which consists of IFN-γ-producing group1 innate lymphoid cell (ILC) lineages (including natural killer cells and ILC1s) (2–4), CD4^+^ type I T helper cells (TH1) (5, 6), and CD8^+^ type I cytotoxic T cells (TC1) mediated responses (7, 8); venoms or helminthes induce type II immune response, which includes IL-4-producing ILC2s (9–11), TH2 cells (6, 12), and TC2 cells (7, 13); and extracellular fungi or bacteria induce type III immune response, which comprises IL-17-producing ILC3s (14, 15), TH17 cells (16–18), and TC17 cells (19, 20). This phenomenon reflects the high plasticity and environmental dependency of immune cells.

As a CD4^+^ helper T cell subset specialized to “help” the germinal center (GC) reaction (21, 22), follicular helper T cells (TFH cells) have been reported to play important roles in type I immune response (23–27), type II immune response (28–32), and type III immune response (33–35). TFH cells express high levels of CXCR5, which is required for their localization in lymphoid follicles (21, 22, 36–40). In the light zone of GCs, they provide crucial signals to antigen-specific B cells and promote somatic hyper mutation, class switch recombination (CSR), and affinity maturation of GC B cells through cellular interactions and cytokine secretion (41–45). In addition, TFH cells also facilitate the differentiation of memory B cells and long-lived plasma cells from GC B cells (21, 22, 46).

TFH cells share similar differentiation processes during different types of immune responses; during the initiation phase of TFH cell differentiation, the expression of some TFs (such as Bcl-6, Ascl2, Maf, and TCF-1) is regulated in certain activated CD4^+^ T cells, which promotes CXCR5 expression (47–51). Next, CXCR5^+^Bcl-6^+^TFH precursor cells migrate to the T-B border zone, where they receive more differentiation signals from activated B cells (52). After this engagement, the reinforced expression of Bcl-6 regulates surface markers, which promote the migration of the TFH cells into GCs, where they provide helper signals to B cells (53, 54). Despite these similarities, TFH cells are also endowed with some unique characteristics for responding to distinct microenvironment associated with different types of microbial infection. Previous studies showed that TFH cells also express lineage-specific TFs like their conventional counterparts when defending against different types of pathogens, such as T-bet, GATA-3, or ROR-γt in type I, II, or III immune responses, respectively (28, 55–60). The production of IFN-γ, IL4, or IL17 driven by these specific TFs in TFH cells can help B cells switch to the optimal class of antibody to clear the microbes (25, 29, 33, 61–66). However, the extent to which TFH cells rely on these TFs for their differentiation or maintenance is not clear.

The transcription factor T-bet was originally discovered as a lineage marker of TH1 cells because it can establish TH1 differentiation and inhibit polarization of other CD4^+^ T cell subsets such as TH2 or TH17 cells (67–69). Later, it was also found to be extensively expressed by multiple different lymphocyte lineages during type I immune response, including both innate and adaptive immune cell subsets (70–72). For example, T-bet has been reported to promote the early differentiation and terminal maturation of NK cells (73–75). It has also been found that T-bet can promote IFN-γ production in ILCs and γδ T cells (4, 76, 77). Moreover, T-bet expressed by DCs can enhance their TH1-priming capacity (78). In NKT cells, T-bet can upregulate CD122 levels and promote survival (74, 79). In addition, T-bet is required for optimal terminal differentiation and granzyme B secretion in CD8^+^ T cells (80, 81). Moreover, T-bet expressed by B cells can promote the survival of memory B cells and enhance IgG2 switching (66, 82). Together, these facts highlight T-bet as the master regulator of type I immune response. In type I immune response, activated antigen-specific CD4^+^ T helper cells mainly differentiate into TH1 and TFH cell subsets (27, 83). Most studies have focused on the role of T-bet in TH1 differentiation and have generally considered T-bet to be a suppressor of TFH differentiation (72, 84–86). However, the exact role of T-bet in the TFH cell response is not well-understood.

In this study, using a combined conditional/inducible knockout system, we investigated the putative role of T-bet in regulating the response of virus-specific TFH cell in acute viral infection. We found the constitutive expression of T-bet in TFH cells during acute viral infection. A great reduction in the magnitude of the TFH cell response was observed when T-bet expression was deficient. Furthermore, microarray analysis showed significant differences in function- and proliferation-related genes between WT and Tbx21^−/−^ TFH cells. In addition, TFH and TH1 cells showed different levels of T-bet dependency in their lineage-specific expression patterns. Thus, our findings demonstrate the crucial and specific role of T-bet in type I TFH cell responses, which suggests that modulation of T-bet expression in TFH cells may be a powerful therapeutic method for the treatment of infectious diseases and autoimmune diseases.

## Materials and Methods

### Mice and Treatment

C57BL/6J (CD45.1^+^ and CD45.2^+^), CD4^cre^ transgenic, Ifng^−/−^ and Tbx21^fl/fl^ mice were purchased from Jackson Laboratory. ERT2^cre^ transgenic mice were kindly provided by Yisong Wan (University of North Carolina). SMARTA (CD45.1^+^) mice were a kind gift from Rafi Ahmed (Emory University). All these strains had a C57BL/6J background. All mice were housed and bred under specific-pathogen-free (SPF) conditions. All mouse experiments were performed following the guidelines of the Institutional Animal Care and Use Committees of Army Medical University. All mice were infected/immunized at 6–10 weeks of age. Lymphocytic choriomeningitis virus (LCMV, Armstrong strain) was provided by Rafi Ahmed (Emory University). A total of 2 × 10^5^ plaque-forming units of LCMV (Armstrong strain) were injected intraperitoneally to establish an acute viral infection model in mice. The *Listeria monocytogenes*-expressing LCMV-gp61-80 was created from vector strain 1. A total of 1 × 10^7^ colony-forming units of recombinant bacteria were injected intravenously to establish a mouse bacterial infection model. NP-KLH (100 μg; N-5060-25; Biosearch Technology) was emulsified 1:1 with Aluminum hydroxide gel (Alum) (21645-51-2; InvivoGen) and was injected subcutaneously to establish a protein immunization model in mice. Tamoxifen (1 mg; T5648; Sigma-Aldrich) was diluted with sunflower oil and injected intraperitoneally into ERT2^cre^-Tbx21^fl/fl^ or ERT2^cre^-Tbx21^fl/fl^ mice to induce gene deletion at the indicated timepoints.

### Flow Cytometry and Antibodies

Stained cells were analyzed by flow cytometry with a FACS Canto II flow cytometer (BD Bioscience). Flow cytometry data were analyzed with FlowJo software (Tree Star). LCMV-GP66 tetramer staining was described previously (51). CXCR5 staining has also been described previously (47). Surface staining was performed in PBS containing 2% fetal bovine serum (weight/volume). Staining for intracellular IgG2c, Bcl2, and IFN-γ was performed using a Cytofix/Cytoperm Fixation/Permeabilization Kit (554714; BD Bioscience). Staining for intranuclear TCF-1, T-bet and FOXP3 was performed with a Foxp3/Transcription Factor Staining Buffer Set (00-5523; eBioscience). For intracellular cytokine production analysis, before surface and intracellular staining, cells were stimulated with GP61-80 peptide for 5 h at 37°C, 5% CO_2_ in the presence of GolgiPlug (BD Bioscience), GolgiStop (BD Bioscience), and DNase I (Sigma-Aldrich). For *in vivo* incorporation of BrdU, mice were given BrdU (1.5 mg of BrdU in 0.5 ml of DPBS) intraperitoneally 3 h before staining. BrdU staining was performed with a BrdU Flow Kit (559619; BD Bioscience) according to the manufacturer's instructions. Annexin V staining was performed with an Annexin V Apoptosis Detection Kit I (559763; BD Bioscience) according to the manufacturer's instructions. The antibodies and reagents used in flow cytometry staining are listed in Table S1.

### Enzyme-Linked Immunosorbent Assay

LCMV-specific IgG and IgG2c were titrated with LCMV lysates and the secondary antibodies HRP-conjugated goat anti-mouse IgG (1036-05; SouthernBiotech) and HRP-conjugated goat anti-mouse IgG2c (1078-05; SouthernBiotech) as previously described (87).

### Adoptive Cell Transfer

To examine the LCMV-specific TFH cell response, 1 × 10^6^ (for analysis before day 3 or after day 30) or 2 × 10^5^ (for analysis between day 3 and day 30) sorted naïve or retrovirus-transduced CD45.1^+^ SMARTA cells (WT or Tbx21^−/−^) were adoptively transferred into naïve or infection-matched CD45.2^+^ mice (WT or Tbx21^−/−^) according to the requirements of the experiments. After being allowed to rest for one day, the cell-transferred hosts were infected intravenously with 2 × 10^6^ plaque-forming units (for analysis at day 3 or earlier) or infected intraperitoneally with 2 × 10^5^ plaque-forming units (for analysis at day 5 or later).

### Mixed Bone Marrow Chimera

To determine the intrinsic role of T-bet, bone marrow cells collected from CD45.2^+^ Tbx21^−/−^ mice and CD45.1^+^ WT mice were mixed at a ratio of 3:7 and transferred intravenously into lethally irradiated (5.5 Gy, twice) naïve WT CD45.1^+^ mice (5 × 10^6^ cells/mouse). After at least 8 weeks of bone marrow reconstitution, the recipients were infected with LCMV.

### Quantitative RT-PCR

To compare gene expression in LCMV-specific TH1 cells and TFH cells differentiated from naïve WT and Tbx21^−/−^ SMARTA cells, SLAM^hi^CXCR5^−^ and SLAM^low^CXCR5^+^ SMARTA cells were sorted from recipient mice and directly lysed with TRIzol LS reagent (10296; Life Technologies). Total RNA was extracted with isopropyl ethanol and reverse-transcribed with a RevertAid H Minus First Strand cDNA Synthesis Kit (K1632; Thermo Scientific). Quantitative PCR of cDNA was carried out with a QuantiNova SYBR Green PCR Kit (208054; Qiagen) on a CFX96 Touch Real-Time System (Bio-Rad). The sequences of *Tbx21* primers used in RT-qPCR are listed here: *Tbx21* (F)-5′ CAATGTGACCCAGATGATCG 3′; *Tbx21*(R)-5′ CAATGTGACCCAGATGATCG 3′. Expression was calculated normalized to *Hprt*.

### Microarray and Analysis

For the isolation of LCMV-specific TH1 cells and TFH cells, SLAM^hi^CXCR5^−^ and SLAM^low^CXCR5^+^ SMARTA cells were sorted from recipient mice adoptively transferred with WT or Tbx21^−/−^ SMARTA cells at day 6 post LCMV infection. For the isolation of naïve CD4^+^ T cells, CD44^−^CD62L^+^CD4^+^ T cells were sorted from naïve WT C57BL/6J mice. The cells were sorted directly into TRIzol LS reagent (10296; Life Technologies). Total RNA was extracted with isopropyl ethanol and submitted to the CapitalBio Corporation for microarray analysis. Gene set enrichment analysis (GSEA) was performed as described previously (88). Clustering analysis was performed and heat maps were constructed using Cluster 3.0 with a hierarchical average linkage method, and the results were visualized using Java TreeView software. Pathway enrichment analysis was performed using KOBAS 3.0 (89).

### Immunofluorescence Staining

Spleen tissues were snap frozen in O.C.T. compound (4583; SAKURA) and stored at −80°C until frozen sectioning. The tissues were cut into 10 μm-thick cryosections and fixed with ice-cold acetone. The sections were rehydrated and blocked with 5% rat serum and 3% BSA with 0.1% Tween and stained with fluorescent-labeled antibodies and reagents, including CD4 (RM4-5; BioLegend), IgD (11-26c.2a; BioLegend), GL7 (GL7; BD Bioscience), and DAPI (R37606; Invitrogen). Images were obtained with an EVOS FL Imaging System (ThermoFisher).

### Statistical Analysis

Statistical analysis was performed with Prism 6.0. Differences between groups were analyzed with paired (for bone marrow chimera experiments) or unpaired two-tailed *t*-tests. A *p*-value <0.05 was considered significant.

## Results

### The Transcription Factor T-Bet Is Selectively Expressed in Type I but not Type II TFH Cells

**Figure 1:**
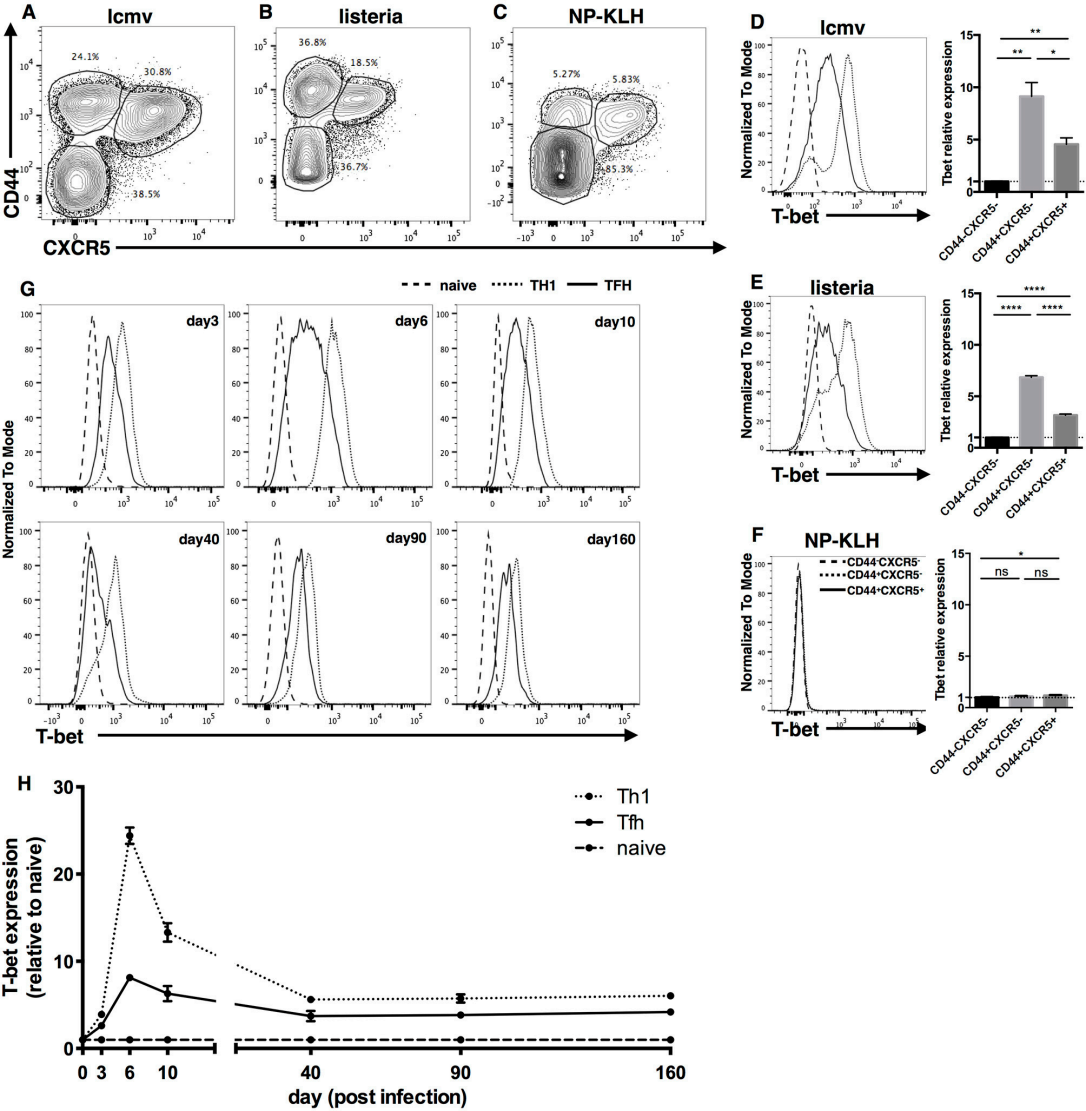
Transcription factor T-bet is selectively expressed in Type I but not Type II TFH cells. **(A–F)** WT C57BL/6 mice were infected with LCMV, LM, or immunized with NP-KLH. Lymphocytes of Spleen (for LCMV and LM infection) or draining lymph nodes (for NP-KLH immunization in alum) were isolated and analyzed for T-bet expression in TFH cells at day 8 post infection/immunization. **(A–C)** Representative flow cytometry of TFH cells (CD44^+^CXCR5^+^), Non-TFH cells (CD44^+^CXCR5^−^) and Naïve CD4^+^ T cells (CD44^−^CXCR5^−^) in LCMV **(A)**, LM **(B)**, infection or NP-KLH **(C)** immunization model. Numbers adjacent to outlined areas indicate percent of each subset in parent subset. **(D–F)** Representative Flow cytometry of T-bet expression in TFH cells, Non-TFH cells and Naïve CD4^+^ T cells (left) and the summary of T-bet expression by calculating the mean fluorescence intensity (MFI) of T-bet in each cell subsets (right) during LCMV **(D)**, LM **(E)** infection, or NP-KLH **(F)** immunization in alum. **(G,H)** WT SMARTA cells (CD45.1^+^) were transferred into WT naïve C57BL/6 mice (CD45.2^+^) and the splenocytes were analyzed for T-bet expression of virus-specific TFH and TH1 cells at day3, 6, 10, 40, 90, and 160 post LCMV infection. **(G)** Representative Flow cytometry of T-bet expression in TFH cells (CD45.1^+^CXCR5^+^), TH1 cells (CD45.1^+^CXCR5^−^) and Naïve CD4^+^ T cells (CD45.2^+^CD44^−^). **(H)** Kinetics of T-bet expression of TFH and TH1 cells by calculating the MFI of T-bet in each subset followed by normalization to the T-bet MFI of Naïve CD4^+^ T cells. ns, not significant; ^*^P < 0.05, ^**^P < 0.01,^****^P < 0.0001 (unpaired two-tailed *t*-test). Data are representative of two independent experiments with 3–5 mice per group (error bars, SEM).

Previously, it has been reported that T-bet is expressed in mouse TFH cells in LCMV infection model (27), which belongs to type I immune response. However, whether TFH cells express T-bet in other type I immune response models or in type II immune responses is not clear. Thus, we first examined the expression of T-bet in *Listeria monocytogenes* (LM) infection, NP-KLH immunization and LCMV infection models. Based on the expression of CD44 and CXCR5, FOXP3^−^CD4^+^ T cells were divided into three subsets, CD44^+^CXCR5^+^, CD44^+^CXCR5^−^, and CD44^−^CXCR5^−^ cells, which were referred to as TFH, non-TFH and naïve CD4^+^ T cells, respectively (Figures 1A–C). At day 8 after immunization, we observed that TFH cells and non-TFH cells generated from the LCMV/LM infection model expressed much higher levels of T-bet than naïve CD4^+^ T cells (Figures 1D,E). In addition, we noticed that TFH cells expressed less T-bet than non-TFH cells in the LCMV/LM infection model (Figures 1D,E), which is consistent with published data (27). However, in the NP-KLH immunization model, there is nearly no detectable T-bet expression in both TFH cells and non-TFH cells (Figure 1F). These data demonstrated that T-bet is selectively expressed in TFH cells derived from type I rather than type II immune responses, suggesting that unlike common transcription factors such as TCF1 or Bcl6, T-bet may be an immune response type-dependent feature of TFH cells.

Next, focusing on the expression of T-bet in type I TFH cells, we investigated the expression kinetics of T-bet in LCMV-specific TFH cells using a SMARTA cell adoptive transfer system. SMARTA cells express LCMV-gp66-specific TCRs, so they can recognize and respond to LCMV and other LCMV-gp66 epitope-carrying microbes (90). We purified naïve ly5.1 SMARTA cells from spleen tissue, transferred them into naïve C57BL/6J recipient mice, and infected the recipient mice with the LCMV strain Armstrong. At different time points post infection, we measured the expression of T-bet in donor SMARTA TH1 and TFH cells. At day 3 post infection, T-bet expression in TFH and TH1 cells was ~2- and 4-fold higher, respectively, than that in naïve CD4^+^ T cells (Figures 1G,H). At day 6 post infection, T-bet expression in TFH and TH1 cells was upregulated to nearly 8- and 24-fold, respectively, compared to that in naïve CD4^+^ T cells (Figures 1G,H). At day 10 post infection, T-bet expression in TFH and TH1 cells had decreased back to levels ~6- and 13-fold higher than those in naïve CD4^+^ T cells (Figures 1G,H). At day 40, 90, and 160 post infection, T-bet expression in TFH and TH1 cells had further decreased and remained ~4- and 6-fold higher, respectively, than that in naive CD4^+^ T cells (Figures 1G,H). Taken together, these data indicate that TFH and TH1 cells share a dynamic similarity in their T-bet expression patterns: T-bet expression sharply increases in the early effect phase, gradually falls back in the contraction phase, and is stably maintained at a certain level in the memory phase. Meanwhile, consistently lower levels of T-bet were observed in TFH cells than in TH1 cells throughout the entire response.

### T-Bet Is Required for TFH Cell Expansion During Acute Viral Infection

**Figure 2:**
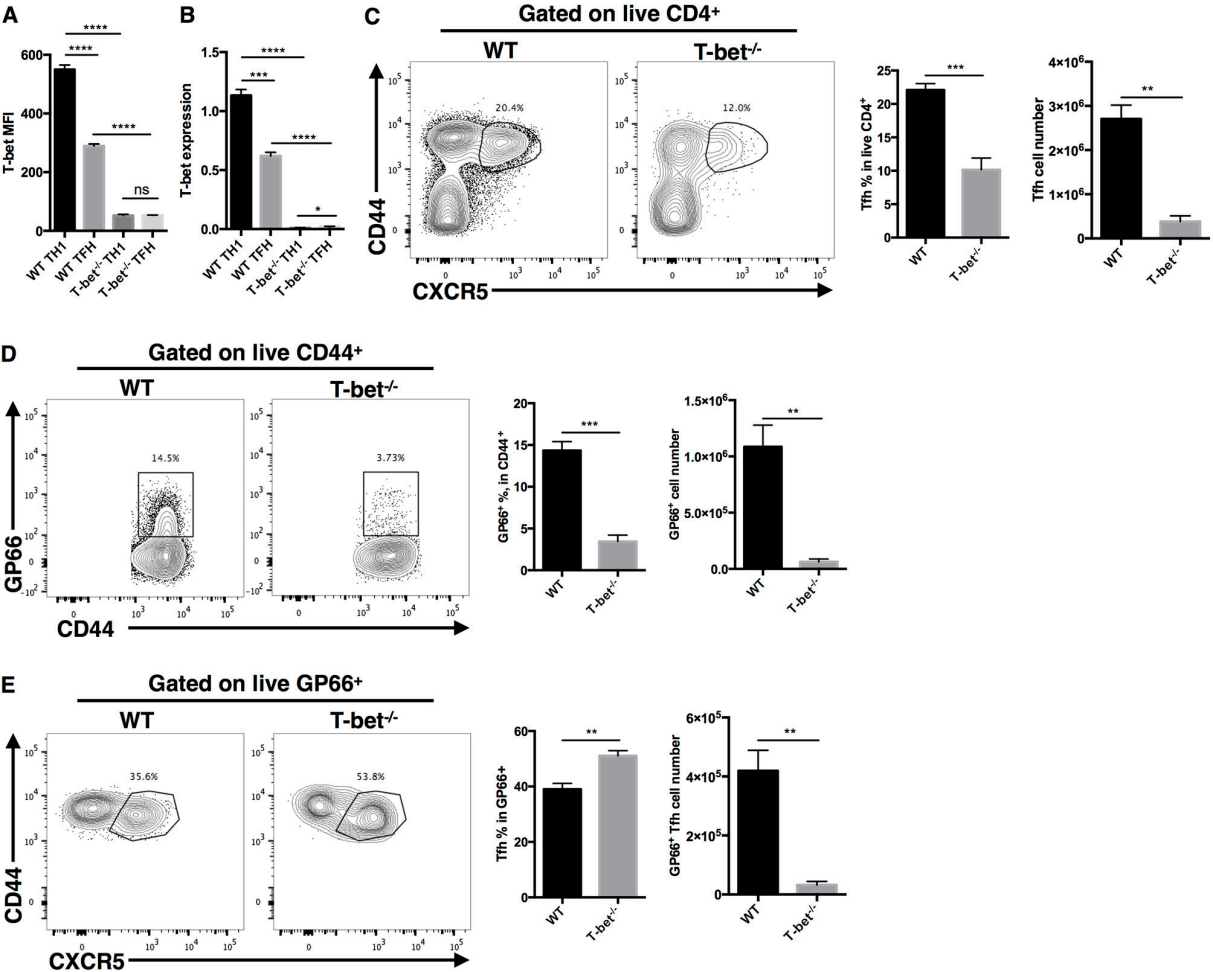
T-bet is required for TFH cell expansion during acute viral infection. WT and Tbx21^−/−^ mice were infected with LCMV. Splenocytes were isolated and analyzed by flow cytometry and RT-qPCR at day 8 post infection. **(A)** Summary of T-bet expression (showed as MFI) of TFH cells (CD44^+^CXCR5^+^) and TH1 cells (CD44^+^CXCR5^−^) in WT and Tbx21^−/−^ mice. **(B)** T-bet gene expression relative to Hprt mRNA of sorted TFH and TH1 cells from WT and Tbx21^−/−^ mice was assessed by RT-qPCR. **(C–E)** Representative Flow cytometry of TFH cells (CD44^+^CXCR5^+^) **(C)**, tetramer-positive CD4^+^ T cells **(D)**, and tetramer-positive TFH cells **(E)** in WT and Tbx21^−/−^ mice (left), and the summary of percentages and number of these cell subsets (right). Numbers adjacent to outlined areas indicate percent of each cell subset in parent subset. ns, not significant; ^*^P < 0.05, ^**^P < 0.01, ^***^P < 0.001, ^****^P < 0.0001 (unpaired two-tailed *t*-test). Data are representative of three independent experiments with 3–5 mice per group (error bars, SEM).

To investigate whether T-bet is required for optimal TFH cell responses during acute viral infection, we generated a CD4^cre^-Tbx21^fl/fl^ strain of mice (called Tbx21^−/−^ mice here) by crossing transgenic CD4^cre^ mice with Tbx21^fl/fl^ mice to selectively knock out the Tbx21 gene (encoding the T-bet protein) in T cells. These Tbx21^−/−^ mice showed normal T cell development *in vivo* (Figures S1A,B). At day 8 post LCMV Armstrong infection, the RT-qPCR and flow cytometry results showed that TH1 and TFH cells from Tbx21^−/−^ mice did not express T-bet (Figures 2A,B). In addition, we observed a significant decrease in the frequency and number of polyclonal CD44^+^CXCR5^+^ TFH cells in the spleens of Tbx21^−/−^ mice (Figure 2C). To determine whether this phenotype was caused by deficient clonal expansion or abnormal differentiation, we used the gp66 tetramer to measure antigen-specific CD4^+^ T cells. The results showed that fewer gp66-specific CD4^+^ T cells were present inTbx21^−/−^ mice (Figure 2D), indicating that clonal expansion of gp66-specific Tbx21^−/−^ CD4^+^ T cells was heavily affected. Consistent with the decreased number of gp66-specific CD4^+^ T cells, the number of gp66-specific TFH cells was also greatly decreased in Tbx21^−/−^ mice (Figure 2E). Furthermore, we observed a mild increase in the frequency of gp66-specific Tbx21^−/−^ TFH cells (Figure 2E), which was consistent with a report that T-bet inhibits TFH cell differentiation *in vitro* (84). Together, these data suggest that T-bet is required for TFH cell response mainly by promoting clonal expansion during acute viral infection.

### Optimal Germinal Center Response Requires T-Bet Expression in TFH Cells

**Figure 3:**
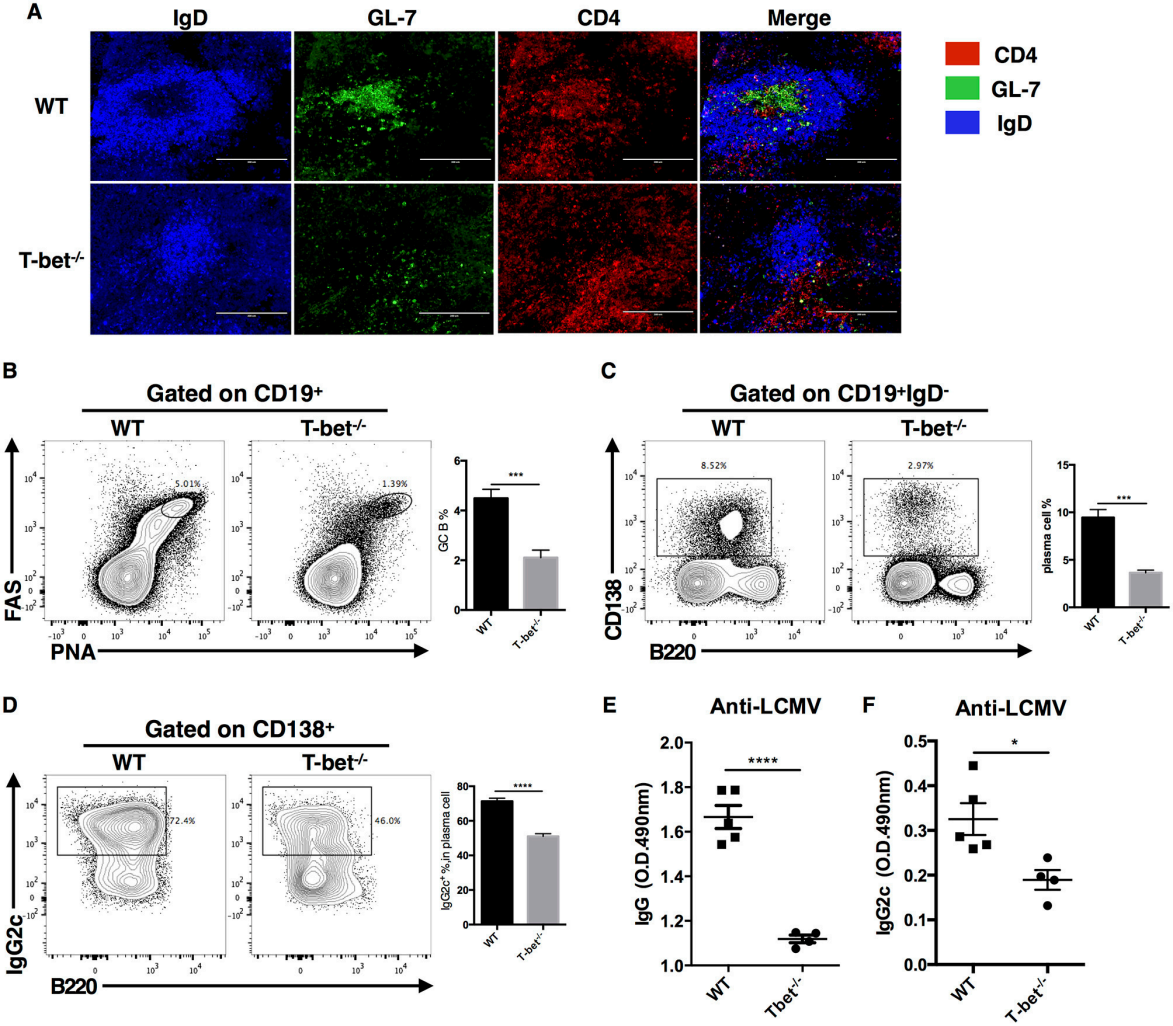
Optimal germinal center response requires T-bet expressed in TFH cell. WT and Tbx21^−/−^ mice were infected with LCMV. Spleens were harvested at day 8 post infection. **(A)** Representative Immunofluorescent staining of splenic B cell follicular with GL-7 (green), anti-IgD (blue), and anti-CD4 (red). **(B,C)** Representative flow cytometry of GC B cells (FAS^+^PNA^+^) **(B)** and plasma cells (CD138^+^B220^low^) **(C)** (left) with its percentages (right) in WT and Tbx21^−/−^ mice. **(D)** Representative flow cytometry of IgG2c^+^ plasma cells (left) with its percentages (right) in WT and Tbx21^−/−^ mice. Numbers adjacent to outlined areas indicate percent of each cell subset in parent subset. **(E,F)** Serum of WT and Tbx21^−/−^ mice were collected and tested for anti-LCMV IgG **(E)** and anti-LCMV IgG2c **(F)** by enzyme-linked immunosorbent assay (ELISA). ^*^P < 0.05, ^***^P < 0.001, ^****^P < 0.0001 (unpaired two-tailed *t*-test). Data are representative of two independent experiments with 3–5 mice per group (error bars, SEM).

Based on the critical role of TFH cells in “helping” GC response, we next investigated whether T-bet deficiency in TFH cells would influence the germinal center response. At day 8 post LCMV Armstrong infection, we observed severely affected GC formation in the spleens of Tbx21^−/−^ mice (Figure 3A). Loss of T-bet expression in TFH cells strongly reduced the frequency of GC B cells and plasma cells (Figures 3B,C). In addition, Tbx21^−/−^ mice showed less IgG2c class switching in plasma cells (Figure 3D) as well as much lower LCMV-specific IgG and subtype IgG2c titers in serum (Figures 3E,F) than WT mice. These data further verified the vital role of T-bet in promoting the TFH cell response and antibody IgG2 class switching.

### T-Bet Is not Required for Type II TFH Cell Response

The observation of a compromised type I TFH response in Tbx21^−/−^ mice during acute viral infection led us to investigate whether T-bet plays an important role in type II TFH cell response. Thus, we tested the TFH and GC responses of Tbx21^−/−^ mice in a protein immunization model. At day 8 post NP-KLH immunization, we observed similar frequencies and numbers of TFH cells in Tbx21^−/−^ mice and control mice (Figure S2A). In addition, we did not find any reductions in the frequency or number of GC B cells as well as plasma cells in Tbx21^−/−^ mice (Figures S2B,C). These results are consistent with the observation that T-bet is not expressed in CD4^+^ T cells during type II immune response. Together with the crucial role of T-bet in regulating type I TFH cell response during acute viral infection, these results confirm that T-bet is an environmentally specific regulator of type I TFH cell response.

### T-Bet Promotes TFH Cell Expansion in a T Cell Intrinsic Manner

**Figure 4:**
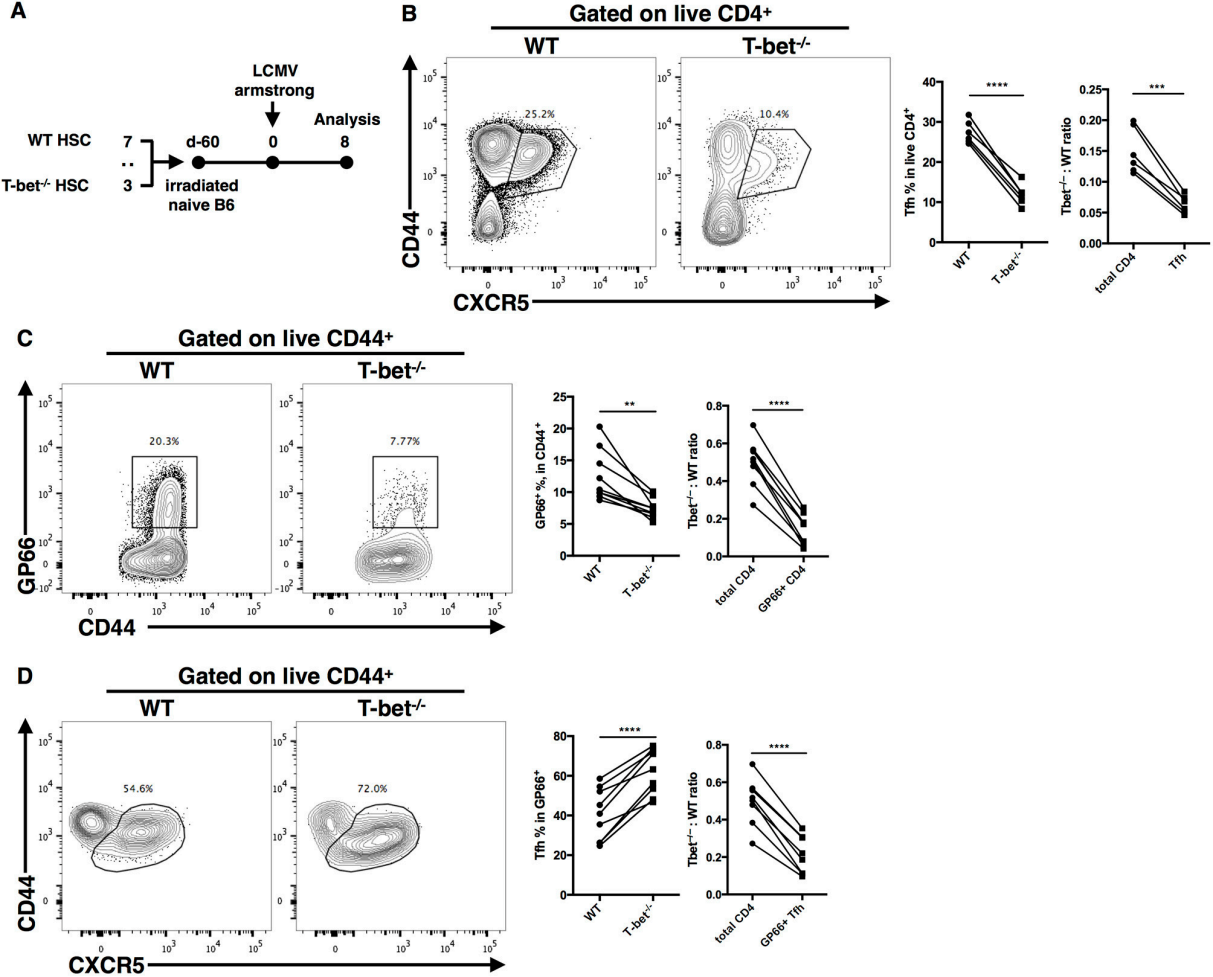
T-bet promotes TFH cell expansion in a T cell intrinsic manner. **(A)** Setup of Bone marrow chimera mice. Bone marrow cells from WT (CD45.1^+^) and Tbx21^−/−^ (CD45.2^+^) mice were mixed in 7:3 ratio and adoptively transferred into lethally irradiated recipient WT (CD45.1^+^) mice. After bone marrow reconstitution, recipients were infected with LCMV (Armstrong strain). Spleens were harvested at day8 post infection. **(B–D)** Representative flow cytometry of TFH cells (CD44^+^CXCR5^+^) **(B)**, tetramer-positive CD4^+^ T cells **(C)** and tetramer-positive TFH cells **(D)** in WT and Tbx21^−/−^. Bone marrow derived cells (left), and the summary of percentages and ratio (by comparing the T-bet^−/−^: WT ratio in target cells and total CD4^+^ T cells) of these cell subsets (right). Numbers adjacent to outlined areas indicate percent of each cell subset in parent subset. ^**^P < 0.01, ^***^P < 0.001, ^****^P < 0.0001 (paired two-tailed *t*-test). Data are representative of two independent experiments with 6 or 9 chimera mice.

In the CD4^cre^-induced Tbx21 knockout system, both CD4^+^ and CD8^+^ T cells lost their capacity to express T-bet. In addition, a deficient GC response might reciprocally amplify the impairment of the TFH cell response in Tbx21^−/−^ mice. To further clarify the cell-intrinsic role of T-bet in regulating the TFH cell response, we set up bone marrow chimeras by reconstituting lethally irradiated WT (ly5.1^+^) recipient mice with a 3:7 ratio mixture of bone marrow cells from Tbx21^−/−^ (ly5.2^+^) and WT (ly5.1^+^) donor mice, respectively (Figure 4A). Chimera mice were infected with LCMV Armstrong after successful bone marrow reconstitution (Figures S3A,B). At day 8 post infection, we still observed a largely decreased frequency of gp66-specific CD4^+^ T cells and polyclonal TFH cells in Tbx21^−/−^ (ly5.2^+^) mice compared to control mice (Figures 4B,C). Similar to what we found in Tbx21^−/−^ mice, the frequency of gp66-specific TFH cells was slightly increased in Tbx21^−/−^ cells of chimera mice (Figure 4D). Moreover, the Tbx21^−/−^: WT ratios of polyclonal TFH cells, gp66-specific CD4^+^ T cells and gp66-specific TFH cells were markedly decreased relative to that of total CD4^+^ T cells (Figures 4B–D). These data confirmed the intrinsic role of T-bet in regulating the TFH cell response during acute viral infection.

### T-Bet Promotes TFH Cell Maintenance by Regulating Proliferation and Apoptosis

**Figure 5:**
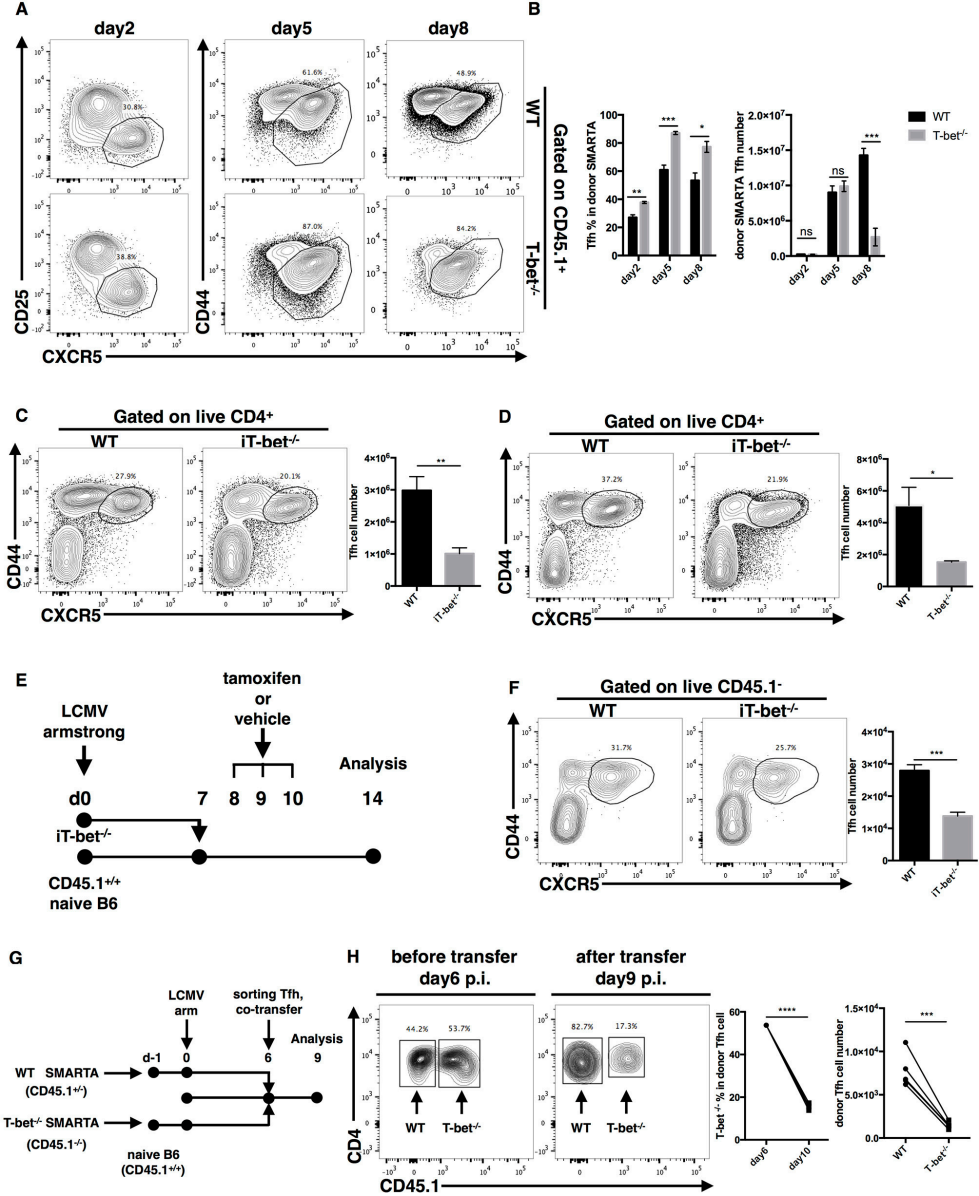
T-bet promotes TFH cell maintenance. **(A,B)** WT or Tbx21^−/−^ SMARTA cells (CD45.1^+^) were transferred into naïve WT mice (CD45.2^+^), which would be infected with LCMV. Splenocytes were isolated and analyzed for TFH response at day2, 5 and 8 post infection. Flow cytometry of TFH cells derived from transferred SMARTA cells **(A)**, and the summary of the percentages and numbers of those cells **(B)**. **(C,D)** WT and iT-bet^−/−^ mice were treated with Tamoxifen for 3 days before or after LCMV infection. Spleens were harvested at day 9 post infection. **(C)** Flow cytometry of TFH cells (left) with its number (right) in mice treat with Tamoxifen before infection (day−3 to−1). **(D)** Flow cytometry of TFH cells (left) with its number (right) in mice treat with Tamoxifen after infection (day 5–7). **(E)** Setup of CD4^+^ T cell transfer experiment. CD4^+^ T cells were purified form spleens of iT-bet^−/−^ mice (CD45.2^+^) without Tamoxifen treatment and adoptively transferred into infection-matched recipient mice (CD45.1^+^). At day 8–10 post infection, recipient mice were treated with Tamoxifen or vehicle. Spleens were harvested at day 14 post infection. **(F)** Representative flow cytometry of TFH cells (left) and summary of TFH cell number (right) in Tamoxifen treated mice (iTbx21^−/−^) and vehicle treated mice (WT) was showed here. **(G)** Setup of TFH co-transfer experiments. WT SMARTA cells (CD45.1^+^CD45.2^+^) and Tbx21^−/−^ SMARTA cells (CD45.1^−^CD45.2^+^) were transferred into naïve B6 mice (CD45.1^+^CD45.2^−^) separately before infecting recipients with LCMV. At day 6 post infection, WT SMARTA TFH cells and Tbx21^−/−^ SMARTA TFH cells were FACS sorted and mixed (about 1:1), then co-transferred into infection-matched B6 mice (CD45.1^+^CD45.2^−^). Spleens were harvested and analyzed for transferred TFH cells at day 9 post infection. **(H)** Flow cytometry of sorted SMARTA TFH cells before (day 6 p.i.) and after (day 9 p.i.) co-transfer (left). The percentage of Tbx21^−/−^ SMARTA TFH cells in total donor TFH cells and the number of WT and Tbx21^−/−^ SMARTA TFH cells at day 9 post infection were summarized (right). Numbers adjacent to outlined areas in **(A,C,D,F,H)** indicate percent of each cell subset in parent subset. ns, not significant; ^*^P < 0.05, ^**^P < 0.01, ^***^P < 0.001, ^****^P < 0.0001 (unpaired **(B–D,F)** or paired **(H)** two-tailed *t*-test). Data in **(C,D,F)** are representative of two independent experiments with 3–5 mice per group (error bars, SEM).

It was clear that the deficiency of the TFH cell response in Tbx21^−/−^ mice was mainly caused by the greatly reduced magnitude of the TFH cell response. To investigate the kinetics of virus-specific TFH cell expansion in Tbx21^−/−^ mice, we transferred the same number of WT or Tbx21^−/−^ SMARTA cells into naïve recipient mice and then infected host mice with LCMV Armstrong. From day 2 post infection, we detected a continuous slightly higher frequency of TFH cells in the Tbx21^−/−^ SMARTA group than in the WT group (Figures 5A,B). At day 2 and day 5 post infection, we did not observe any differences in the numbers of Tbx21^−/−^ and WT SMARTA TFH cells (Figures 5A,B). To our surprise, the number of Tbx21^−/−^ SMARTA TFH cells decreased sharply at day8 post infection (Figures 5A,B). Besides, the reduction in the virus-specific TFH cell population in Tbx21^−/−^ mice might not have been caused by impaired early activation of CD4^+^ T cells (Figures S4A,B). Taken together, the results suggest the possibility that the loss of TFH cells in Tbx21^−/−^ mice was mainly caused by reduced maintenance at the late phase of the anti-viral immune response.

To more carefully investigate the influence of T-bet on TFH cell maintenance at the late phase of infection, we generated ERT2^cre^-Tbx21^fl/fl^ mice (iTbx21^−/−^) by crossing Tbx21^fl/fl^ mice with ERT2^cre^ transgenic mice, in which Tbx21 gene knockout could be induced by tamoxifen treatment. We treated mice with tamoxifen at 1–3 days before or 5–7 days after LCMV infection to induce T-bet deletion before or after TFH cell commitment, respectively. Under both of these circumstances, we observed a lower abundance of TFH cells in iTbx21^−/−^ mice at day 9 post infection (Figures 5C,D). In addition, iTbx21^−/−^ CD4^+^ T cells were purified and adoptively transferred into infection-matched recipient mice at day 7 post LCMV infection (Figure 5E). After 3 days of tamoxifen or vehicle administration (days 8–10), we observed a decreased number of donor TFH cells in mice treated with tamoxifen than in control mice at day 14 post infection (Figure 5F). These results suggest that T-bet is required for the TFH cell response even after TFH commitment.

Furthermore, to investigate the sharp decrease in the number of Tbx21^−/−^ SMARTA TFH cells at the late effector phase, we sorted WT and Tbx21^−/−^ SMARTA TFH cells from recipient mice at day 6 post LCMV Armstrong infection and adoptively transferred a 1:1 ratio mixture of WT and Tbx21^−/−^ SMARTA TFH cells into infection-matched mice (Figure 5G). At day 9 post infection, we observed that the ratio of WT and Tbx21^−/−^ SMARTA TFH cells had changed to ~4:1 (Figure 5H). Taken together, these results indicated that intrinsic expression of T-bet is essential for TFH maintenance at the late effector phase.

**Figure 6:**
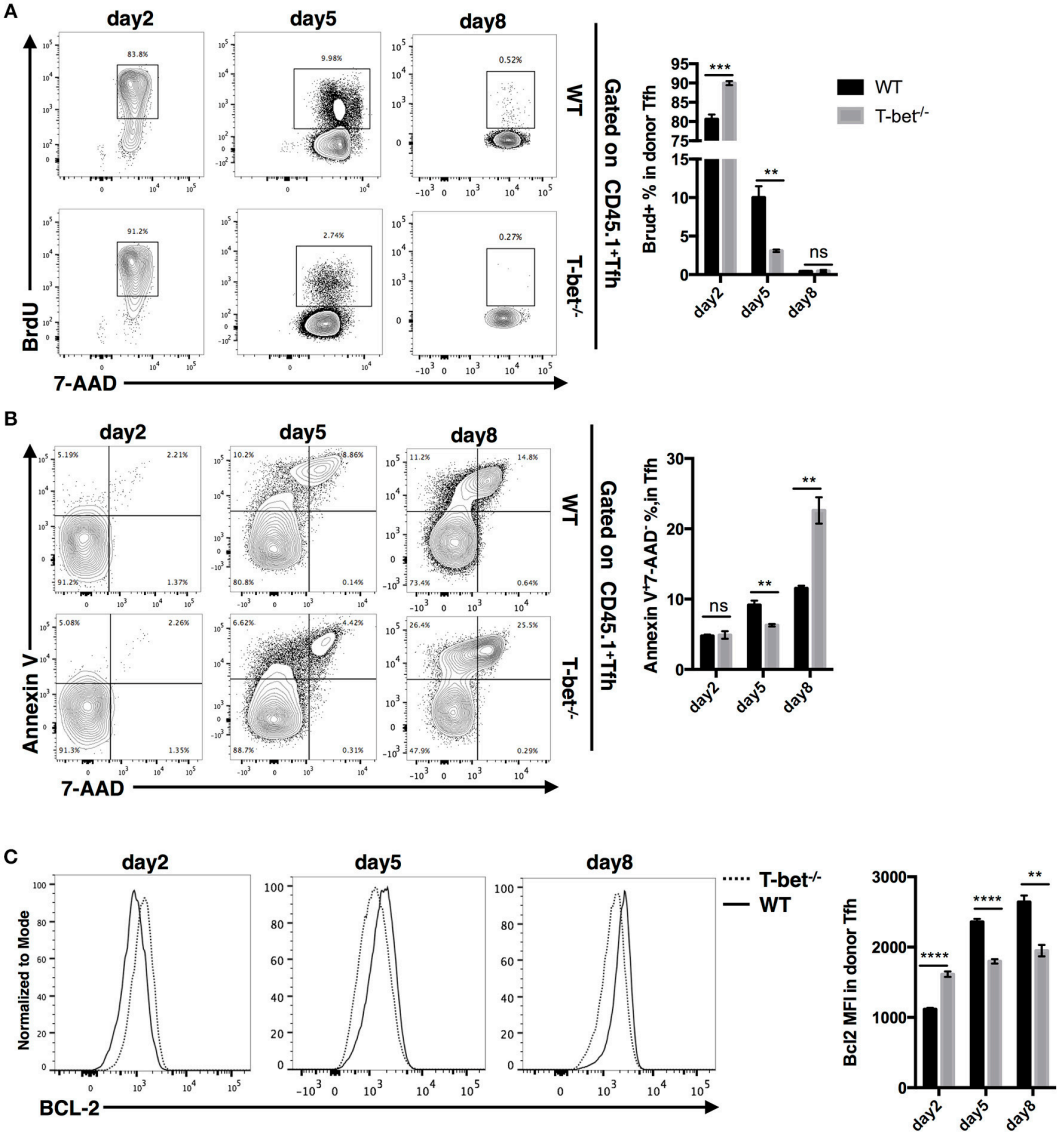
T-bet regulates TFH cell proliferation and apoptosis. WT or Tbx21^−/−^ SMARTA cells (CD45.1^+^) were transferred into naïve WT mice (CD45.2^+^), which would be infected with LCMV. Splenocytes were isolated and analyzed for TFH response at day 2, 5, and 8 post infection. **(A)** Flow cytometry of BrdU incorporation in TFH cells (left), and summary of BrdU-incorporated cells percentage (right). Numbers adjacent to outlined areas indicate percent of each cell subset in parent subset. **(B)** Flow cytometry of Annexin-V and 7-AAD staining in TFH cells (left), and summary of Annexin-V^+^7-AAD^−^ percentage (right). Numbers in quadrants indicate percent cells in each. **(C)** Flow cytometry of BCL-2 expression in TFH cells (left), and the summary of Bcl-2 expression (showed as MFI) of TFH cells. ns, not significant; ^**^P < 0.01, ^***^P < 0.001, ^****^P < 0.0001 (unpaired two-tailed *t*-test). Data are representative of two independent experiments with 3–5 mice per group (error bars, SEM).

Next, to gain insight into the reason for the reduction in the TFH cell population, we measured the proliferation and apoptosis of SMARTA TFH cells. At day 2 post infection, we observed even higher proliferation and expression of the survival marker Bcl2 but comparable apoptosis in Tbx21^−/−^ SMARTA TFH cells compared to WT cells (Figures 6A–C). However, at day 5 post infection, the proliferation rate of Tbx21^−/−^ SMARTA TFH cells dropped quickly to a significantly lower level than that of WT cells (Figure 6A), which was consistent with the lower Bcl2 expression in Tbx21^−/−^ SMARTA TFH cells (Figure 6C). At day 8 post infection, higher apoptosis rates and lower Bcl2 expression were found in Tbx21^−/−^ SMARTA TFH cells than in WT cells (Figures 6B,C). These results suggested that T-bet controls the TFH cell maintenance ultimately by promoting proliferation at the mid phase and inhibiting apoptosis at the late effector phase.

### T-Bet Dependency of the TFH and TH1 Cell Transcriptomes

**Figure 7:**
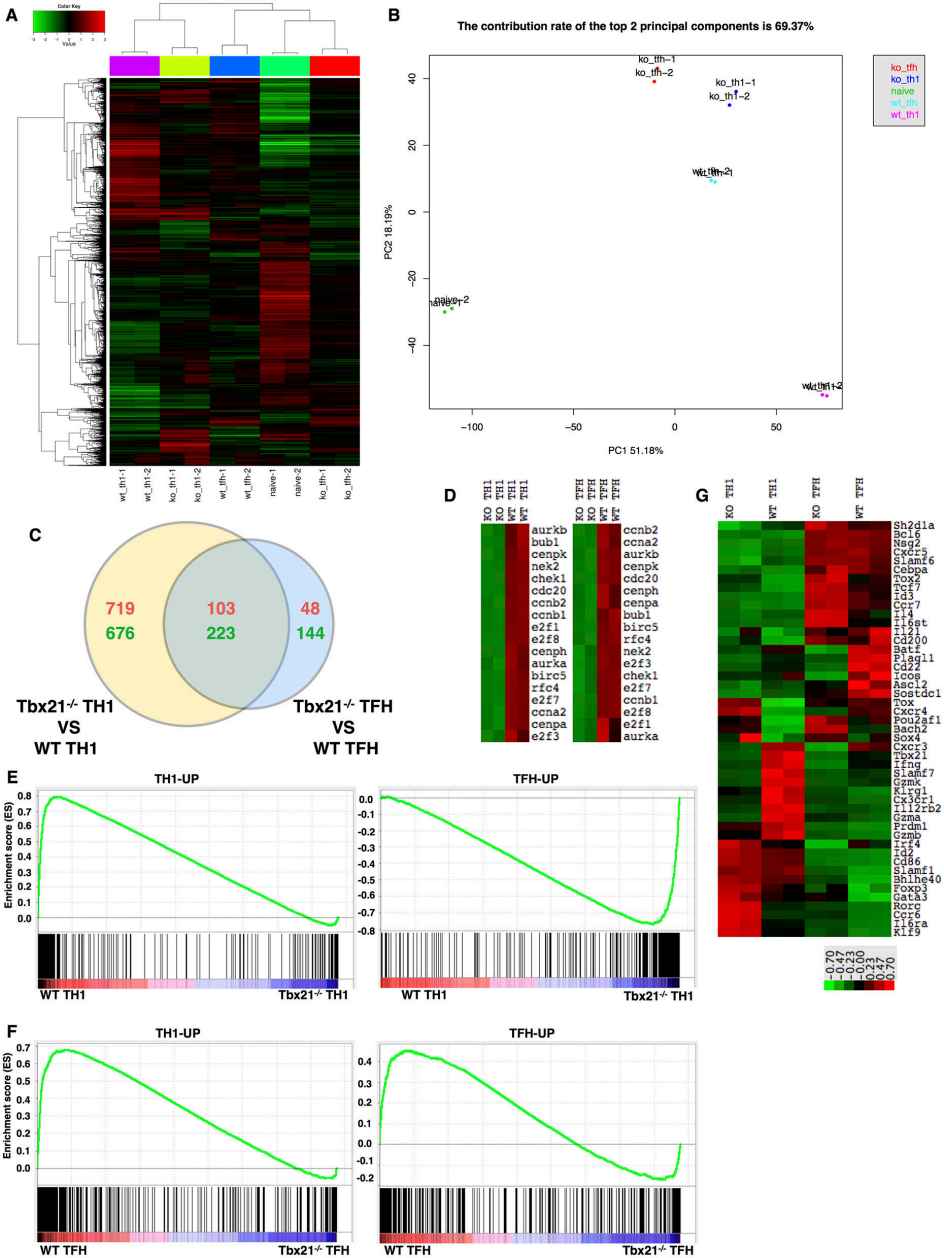
T-bet dependency of the TFH and TH1 cell transcriptomes. **(A)** Clustering of genes based on their expression in WT TH1, WT TFH, Tbx21^−/−^ TH1, and Tbx21^−/−^ TFH cells. **(B)** Principal component analysis (PCA) of genes in the five cell populations listed in **(A)**. **(C)** Venn diagram comparing the differentially expressed genes in TH1 and TFH cells. The red numbers represent upregulated genes, and the green numbers represent downregulated genes. **(D)** Heat map of genes related to cell proliferation in WT TH1, Tbx21^−/−^ TH1, WT TFH, and Tbx21^−/−^ TFH cells. **(E)** GSEA of WT and Tbx21^−/−^ TH1 genes showing gene sets upregulated in TH1 cells relative to TFH cells (left) and gene sets upregulated in TFH cells relative to TH1 cells (right). **(F)** GSEA of WT and Tbx21^−/−^ TFH genes showing gene sets upregulated in TH1 cells relative to TFH cells (left) and gene sets upregulated in TFH cells relative to TH1 cells (right). The gene sets used in GSEA include published data (GEO accession code GSE21379) and our microarray data (GEO accession code GSE122931). **(G)** Heat map of genes related to T cell differentiation and function in WT TH1, Tbx21^−/−^ TH1, WT TFH, and Tbx21^−/−^ TFH cells.

To investigate the molecular mechanisms regulated by T-bet in TFH and TH1 cell response, we sorted WT and Tbx21^−/−^ SMARTA TFH and TH1 cells from recipient mice at day 6 post LCMV Armstrong infection after adoptive transfer, as well as naïve mice-derived CD4^+^ T cells, for gene expression profile analysis. The gene expression patterns differed greatly between WT and Tbx21^−/−^ cell population at the genome-wide level (Figures 7A,B). We observed 822 upregulated and 899 downregulated genes in Tbx21^−/−^ TH1 cells relative to WT TH1 cells (Figure S5A). Accordingly, we identified 151 upregulated and 367 downregulated genes in Tbx21^−/−^ TFH cells (Figure S5B). Among these differentially expressed genes, 103-up and 223-down regulated genes were shared by Tbx21^−/−^ TFH and TH1 cells (Figure 7C). Besides, PANTHER pathway enrichment analysis of the changed genes in Tbx21^−/−^ TFH and TH1 cells also showed similar enrichment in many important pathways, such as DNA replication, Apoptosis, and Interferon-gamma signaling pathway, which account for the impaired maintenance of Tbx21^−/−^ TFH cells (Figures S5C,D). To further figure out the downstream factors involved in the maintenance of differentiated TFH and TH1 cells controlled by T-bet, we did the gene set enrichment analysis focusing on cell proliferation and survival and found a reduction to a similar extent in an array of proliferation and survival relevant genes, such as *Ccna2, Ccnb2, Aurkb, E2f1, E2f7*, and *E2f8* in both Tbx21^−/−^ TH1 and TFH cells compared to their WT counterpart (Figure 7D), highlighting the shared regulatory pathway important for both TFH and TH1 proliferation and survival that is likely imprinted by the same Type-I microenvironment (58).

Despite these similarities, bioinformatics analysis of the microarray data also observed many differences in the dependency of T-bet in TFH and TH1 cell development. Principal component analysis (PCA) showed that after T-bet deletion, the gene expression profile of Tbx21^−/−^ TH1 cells shifted toward that of TFH cells to some extent (Figure 7B). Then, we selected sets of genes that were upregulated in TFH cells (TFH-UP) compared with non-TFH cells or upregulated in TH1 cells (TH1-UP) compared with TFH cells based on both published data (91) (GEO accession code GSE21379) and our microarray data (GEO accession code GSE122931). Gene set enrichment analysis (GSEA) of TH1 cells showed that the TH1-UP gene set was enriched in WT TH1 cells, whereas the TFH-UP gene set was enriched in Tbx21^−/−^ TH1 cells, which suggested to some extent that the gene-expression pattern of Tbx21^−/−^ TH1 cells lost TH1 signature and change to TFH signature (Figure 7E). Similar to that of TH1 cells, GSEA of TFH cells showed that the TH1-UP gene set was enriched in WT TFH cells (Figure 7F). However, different from what we have observed in TH1 cells, genes in the TFH-UP gene set were enriched in WT TFH cells much more than in Tbx21^−/−^ TFH cells, which suggested that the gene-expression pattern of Tbx21^−/−^ TFH cells lost TH1 signature but also lost part of its TFH signature (Figure 7F). Through further assessed 48 genes known to be associated with T cell differentiation or function, we found a distinct T-bet dependency of the TFH and TH1 cell gene-expression pattern (Figure 7G). In TH1 cells, We found that the transcription levels of a set of important Th1 lineage associated genes like *Il12rb2* (92) *Cxcr3, Prdm1, Gzmb, Gzmk, Gzma, Cx3cr1*, and *Ifng* were significantly decreased in Tbx21^−/−^ Th1 cells than in WT TH1 cells. We also observed *Prdm1* expression was lower in Tbx21^−/−^ TH1 cells than in WT TH1 cells. In addition, the expression of *Foxp3, Gata3*, and *Rorc*, which are essential for Treg, TH2, and TH17 cell differentiation, respectively, was higher in Tbx21^−/−^ TH1 cells than in WT TH1 cells (Figure 7G). On the other hand, the abundances of TFH lineage-specification associated genes, including *Tox2, Id3, Bhlhe40, Il6st, Il6ra*, and *Tcf7* were much increased in Tbx21^−/−^ TFH cells (Figure 7G), at least in part explaining the differential role of T-bet in regulating the program of TH1 and TFH differentiation. We compared the expression of *Batf4* (93) and *Irf4* in WT and Tbx21^−/−^ TFH cells. As expected, we observed the dramatically decrease expression of *Batf* and *Irf4* in Tbx21^−/−^ TFH cells. Besides, the significant lower expression of *Icos* (94) was also detected in Tbx21^−/−^ TFH cells compared to that in WT counterparts (Figure 7G). Whereas, expression of other TFH cell-relevant genes like *Prdm1, Bcl6*, and *Cxcr5* was not significantly influenced by T-bet deletion (Figure 7G). The imbalanced impact of T-bet deletion on TH1 and TFH cells may interpret the mildly higher frequency of TFH cells than TH1 cells in antigen-specific CD4^+^ T cells that we observed (Figures 2E, 4D, 5A). Together, these data indicate both similarities and differences in transcriptome dependency on T-bet in TFH and TH1 cells during acute viral infection.

### IFN-γ as a Candidate Downstream Target of T-Bet in Regulating TFH Cell Expansion

**Figure 8:**
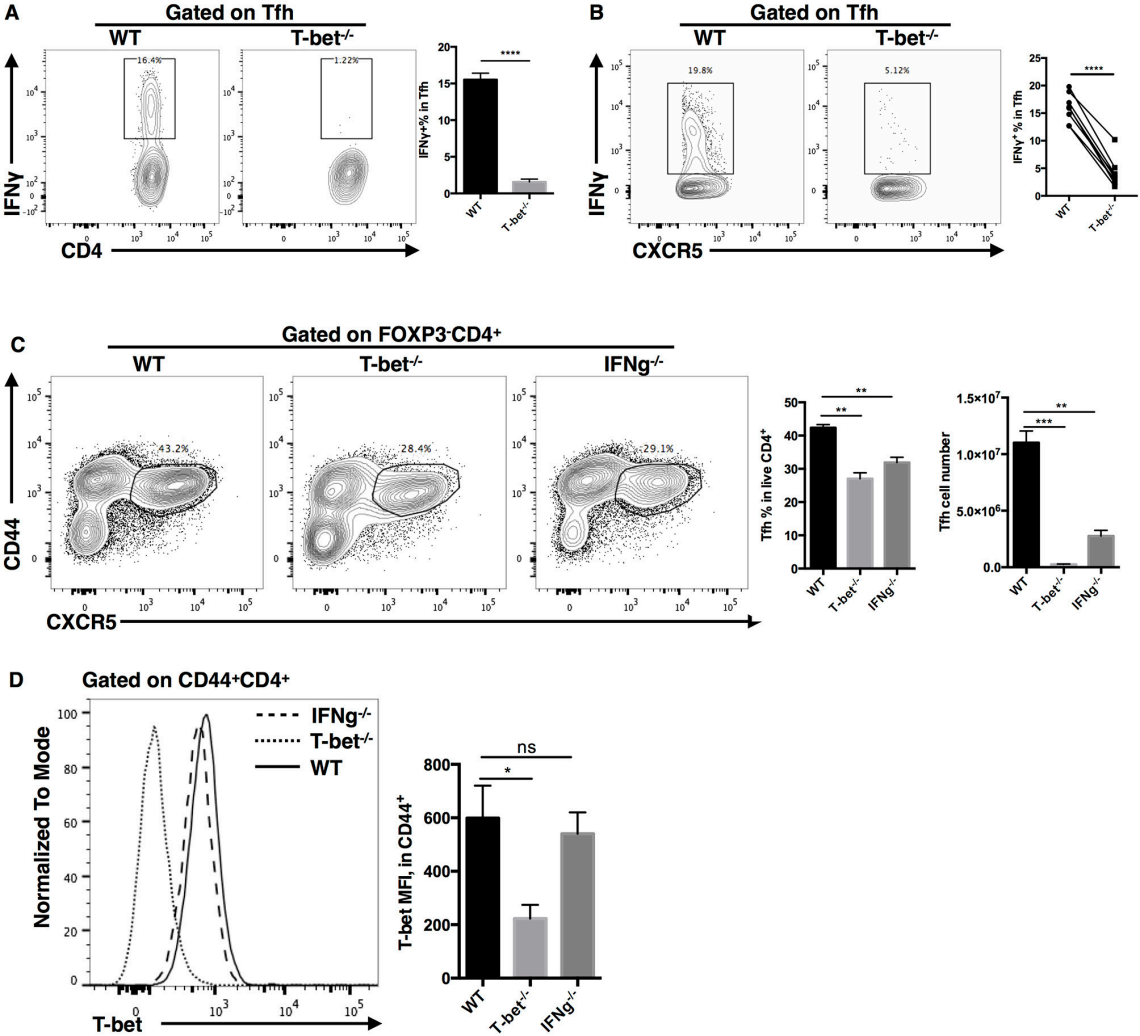
IFN-γ as a candidate downstream target of T-bet in regulating TFH cell expansion. **(A)** Flow cytometry of IFN-γ production in TFH cells (left), with the summary of IFN-γ percentages (right) derived from WT and Tbx21^−/−^ mice at day 8 post LCMV infection. **(B)** Flow cytometry of IFN-γ production in WT and Tbx21^−/−^ TFH cells (left), with the summary of IFN-γ percentages (right) derived from same bone marrow chimera mice at day 8 post LCMV infection. **(C,D)** WT, Tbx21^−/−^ and Ifng^−/−^ mice were infected with LCMV. Spleens were harvested at day 8 post infection. **(C)** Flow cytometry of TFH cells (left) with the summary of percentages and numbers (right) in these mice. **(D)** Flow cytometry of T-bet expression (left), and the summary of T-bet expression (showed as MFI) in TFH cells of WT, Tbx21^−/−^, and Ifng^−/−^ mice. Numbers adjacent to outlined areas in **(A–C)** indicate percent of each cell subset in parent subset. ns, not significant; ^*^P < 0.05, ^**^P < 0.01, ^***^P < 0.001, ^****^P < 0.0001 (unpaired two-tailed *t*-test). Data are representative of two independent experiments with 3–5 mice per group (error bars, SEM).

As a direct target of T-bet, it has been reported that IFN-γ could promote clonal expansion and survival of CD4^+^ T cells (95). Microarray analysis also showed that the interferon-gamma signaling pathway was severely impaired in TFH cells after T-bet deletion. In addition, we observed largely decreased IFN-γ production in *ex vivo* recalled Tbx21^−/−^ TFH cells (Figures 8A,B). To investigate if IFN-γ could regulate TFH cell expansion, we compared the TFH cell responses in Tbx21^−/−^ and Ifng^−/−^ mice during LCMV Armstrong infection. At day 8 post infection, we found that the frequency and number of TFH cells were decreased in both Tbx21^−/−^ and Ifng^−/−^ mice (Figure 8C). In addition, T-bet expression was not affected by IFN-γ deletion (Figure 8D). Although not sufficient, these results suggest that IFN-γ might be a candidate target of T-bet in regulating TFH cell expansion.

## Discussion

Follicular helper T cells (TFH cells) play critical roles in type I, II, and III immune responses, but how TFH cells adapt to different environments has remained largely unknown. In this study, we identified a key link between the transcription factor T-bet and type I TFH cell response during acute viral infection. We found that T-bet is specifically expressed in TFH cells originating from type I but not type II immune response. Tbx21^−/−^ mice exhibited significant deficiency in the TFH cell response during acute viral infection. We observed a greatly decreased magnitude of the TFH cell response in Tbx21^−/−^ mice compared to WT mice, although a slightly increased ratio of TFH cells was observed. Based on these results, we concluded that T-bet is required for optimal type I TFH cell response.

In addition to LCMV infection, type I TFH cells have also been discovered as TH1-biased TFH cells in simian immunodeficiency virus infection and TH1-polarized TFH cells in malaria infection (96, 97). Type I TFH cells not only express Bcl6, CXCR5, IL21, and PD-1 but also coexpress CXCR3 and IFN-γ. The secreted IFN-γ can help antibodies from B cells switch to the IgG2a/c class, which is essential for efficient elimination of viruses and other pathogens. Similar to the case in TH1 cells, the expression of TH1-associated molecules in type I TFH cells is also induced by the transcription factor T-bet. In addition, our research demonstrated the crucial role of T-bet in promoting TFH cell proliferation and maintenance. Based on the specialized role of type I TFH cells in defending against intracellular pathogens, we propose that this Th1-like effector TFH population be named TFH1.

During TH1 differentiation, it has been reported that T-bet attenuates the TFH cell-like phenotype in the late phase of TH1 specification by repressing the expression of Bcl6 and other molecules associated with TFH cell development (84). In addition, T-bet has been found to inhibit Tcf7 expression by directly binding with the Tcf7 gene promoter and suppress Bcl6 function by physically interacting with the Bcl6 protein (85, 86, 98). These *in vitro* results suggest that T-bet uses multiple mechanisms to inhibit TFH differentiation. However, evidence supporting the role of T-bet in regulating the TFH phenotype is not sufficient at the *in vivo* level. In our study, we clearly showed that the maintenance of TFH at the later effector phase is sharply impaired after T-bet deletion even though mildly increased early TFH differentiation was observed. Additionally, the constitutive expression of T-bet in type I TFH cells may suppress early TFH generation but sustain the clonal expansion of TFH cells at the late stage. Thorough understanding of the distinct role of T-bet in type I TFH cells at different stages necessitates further investigation in the future.

Notably, we did not observe any T-bet expression in type II TFH cells during protein immunization. Accordingly, Tbx21^−/−^ mice showed normal TFH and GC responses during protein immunization. These results remind us that, unlike Bcl6 or Blimp1, the transcription factor T-bet is not a fundamental regulator of “all-weather” TFH cell responses under natural conditions. In other words, T-bet is a type I TFH cell-specific regulator, suggesting that diverse transcription factors are required for optimal TFH cell responses in different environments.

In addition, our microarray analysis results showed that there are not only many similar but also many different important changes in the TFH and TH1 cell transcriptomes that occur in a T-bet-dependent manner. On the one hand, Tbx21^−/−^ TFH cells share many altered genes with TH1 cells, including genes enriched in signaling pathways involved in DNA replication, apoptosis and interferon-gamma signaling. On the other hand, many TFH differentiation-related genes were altered in different directions and to different degrees in Tbx21^−/−^ TH1 and TFH cells. Four possible mechanisms might be involved in this scenario. First, some genes are regulated by T-bet in a redundant way, which means that the regulatory role of T-bet may be unnecessary if these genes have already been up- or downregulated by other transcription factors. Second, differences in chromatin accessibility between TFH and TH1 cells would lead to differences in the binding affinity of T-bet. Third, by interacting with different transcription factors, T-bet could differentially regulate gene expression in TFH and TH1 cells. Fourth, the post transcriptional modification of T-bet is different in TFH and TH1 cells, which might result in different or even opposite regulatory functions at the same gene loci. Further studies are needed to explore the exact mechanism underlying the contradictory effects of T-bet in regulating the development of TH1 and TFH cells.

Overall, this study revealed that T-bet, although slightly inhibiting TFH differentiation, mainly supports type I TFH cell response by promoting cell proliferation and apoptotic intervention to maintain the TFH cell response at the late effector phase during acute viral infection. These findings provide important insights into the transcription factor-mediated regulation of the environmental suitability of TFH cells.

## Data Availability

The datasets generated for this study can be found in Gene Expression Omnibus, GSE122931.

## Ethics Statement

All mouse experiments were performed following the guidelines of the Institutional Animal Care and Use Committees (IACUCs) of Army Medical University. The protocols were approved by the IACUCs.

## Author Contributions

PW, YC, YuW, and LY designed and supervised the study. PW, YW, LuX, MX, JW, QH, BL, XC, LiX, SY, and YH performed experiments. PW wrote the manuscript with YW. QB and RH helped with analysis. All authors read and approved the manuscript.

### Conflict of Interest Statement

The authors declare that the research was conducted in the absence of any commercial or financial relationships that could be construed as a potential conflict of interest.
