# Supplementary material for: The Transcription Factor T-Bet Is Required for Optimal Type I Follicular Helper T Cell Maintenance During Acute Viral Infection
Source: Front Immunol. 2019 Mar 29;10:606. doi: 10.3389/fimmu.2019.00606 (PMC6449430; doi:10.3389/fimmu.2019.00606)
Supplement: Supplementary file 1 [file Data_Sheet_1.docx]

Supplementary Material

The Transcription Factor T-bet is Required for Optimal Type I Follicular Helper T Cell Maintenance during Acute Viral Infection

Pengcheng Wang^1,2,#^,Youping Wang^1,#^, Luoyingzi Xie^1,#^, Minglu Xiao^1^, Jialin Wu^1^, LifanXu^1^, QiangBai^1^, YaxingHao^1^, Qizhao Huang^4^, Xiangyu Chen^1^, Ran He^1^, Baohua Li^1^, Sen Yang^3^, Yaokai Chen^3,^*,Yuzhang Wu^1,^*and Lilin Ye^1,^*

^1^Institute of Immunology, PLA,Third Military Medical University, Chongqing, China

^2^National Clinical Research Center of Kidney Diseases, Jinling Hospital, Nanjing, China

^3^Chongqing Public Health Medical Center, Chongqing, China

^4^Cancer Center, The General Hospital of Western Theater Command, Chengdu, China

^#^These authors contributed equally to this work

***Correspondence:**

Yaokai Chen
yaokaichen@hotmail.com

Yuzhang Wu
wuyuzhang@tmmu.edu.cn

Lilin Ye
yelilinlcmv@tmmu.edu.cn

**Supplementary Table**

**Supplementary Table 1 | Antibodies and Reagents**

| Antibody/Reagent | Clone/Cat.No. | Company |
| --- | --- | --- |
| CD4 | RM4-5 | Biolegend |
| CD8 | 53-6.7 | BD Biosciences |
| B220 | RA3-6B2 | eBioscience |
| CD19 | 6D5 | Biolegend |
| CD44 | IM7 | eBioscience |
| CD69 | H1.2F3 | Biolegend |
| CD25 | PC61.5 | Biolegend |
| T-bet | 4B10 | Biolegend |
| Foxp3 | FJK-16s | eBioscience |
| CXCR5 | 2G8 | BD Bioscience |
| TNFa | MP6-XT22 | Biolegend |
| IL2 | JES6-5H4 | Biolegend |
| IFNγ | XMG1.2 | BD Bioscience |
| SLAM | TC15-12F12.2 | Biolegend |
| Bcl6 | K112-91 | BD Bioscience |
| TCF1 | C46C7 | CST |
| PNA | FL-1071 | Vector Labs |
| FAS | JO2 | BD Bioscience |
| CD138 | 281-2 | BD Bioscience |
| Ki67 | B56 | BD Bioscience |
| Bcl2 | 51-15025X | BD Bioscience |
| Caspase3 | 51-68654X | BD Bioscience |
| CD45.1 | A20 | Biolegend |
| CD45.2 | 104 | Biolegend |
| Va2 | B20.1 | Biolegend |
| CD127 | A7R34 | Biolegend |
| CD62L | MEL-14 | eBioscience |
| PD-1 | RMP1-30 | eBioscience |
| GITR | DTA-1 | eBioscience |
| CD71 | R17217 | eBioscience |
| CD98 | RL388 | eBioscience |
| IgG2c | 130-097-950 | MiltenyiBiotec |
| IgD | 11-26c.2a | BD Bioscience |
| Tim3 | RMT3-23 | Biolegend |
| Annexin V | 51-65875X | BD Bioscience |
| Brdu | 51-23619U | BD Bioscience |
| FC-blocker | NB309 | Innovex Biosciences |
| KLRG1 | 2F1 | BD Bioscience |
| GL-7 | GL7 | BD Bioscience |

## Supplementary Figures


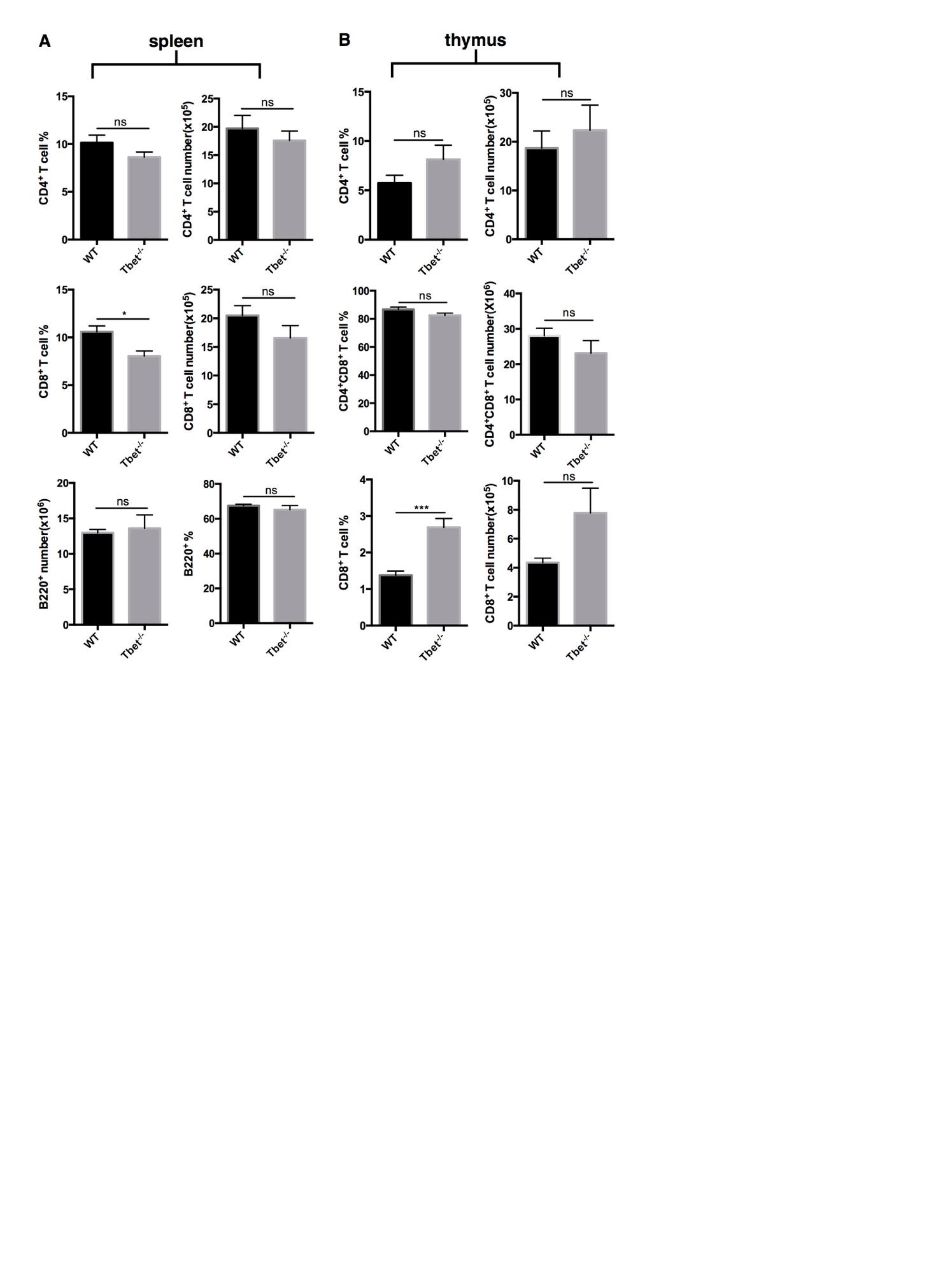


**S FIGURE 1 | Naive state of CD4/CD8+T cells in Tbx21^-/-^ mice (associated with Figure 2).** Spleen as well as thymus were harvested from Naïve WT and Tbx21^-/-^ mice and analyzed for naïve state of lymphocytes. **(A)** Summary the percentages and numbers of CD4^+^ T cells, CD8^+^ T cells and B220^+^ B cells in spleen. **(B)** Summary the percentages and numbers of CD4^+^ T cells, CD8^+^ T cells and CD4^+^CD8^+^ T cells in thymus. ns, not significant; **P* < 0.05, ****P* < 0.001 (unpaired two-tailed *t*-test). Data are representative of two independent experiments with 3-5 mice per group (error bars, SEM).

**
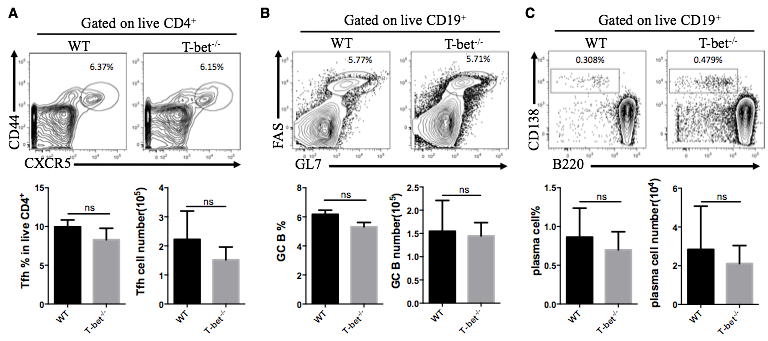
**

**S FIGURE 2 | T-bet is not required for Type II TFH cell response.** **(A-C)** WT and Tbx21^-/-^ mice were immunized with NP-KLH. Draining lymph nodes were harvested at day8 post infection. Representative flow cytometry of TFH cells (CD44^+^CXCR5^+^) **(A)**, GC B cells (FAS^+^PNA^+^) **(B)** and plasma cells (CD138+B220^low^) **(C)** (up) with its percentages and numbers (below) in WT and Tbx21^-/-^ mice. Numbers adjacent to outlined areas indicate percent of each cell subset in parent subset. ns, not significant; **P* < 0.05, ***P* < 0.01, ****P* < 0.001 (unpaired two-tailed *t*-test). Data are representative of two independent experiments with 3-5 mice per group (error bars, SEM).


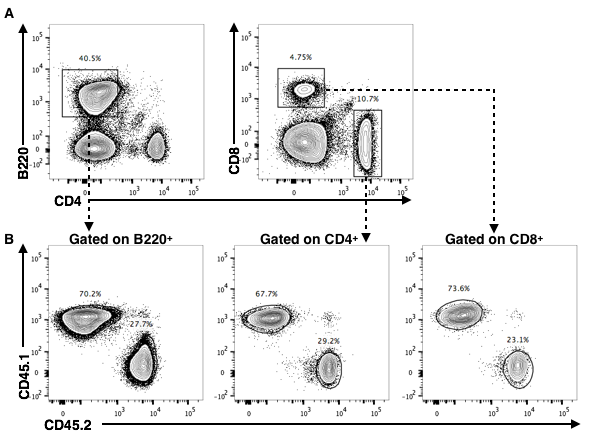


**S FIGURE 3 | successful reconstitution of bone marrow chimera mice (associated with Figure 4).** Bone marrow cells collected from CD45.2^+^ Tbx21^-/-^ mice and CD45.1^+^ WT mice were mixed at a ratio of 3:7 and transferred intravenously into lethally irradiated (5.5 Gy, twice) naïve WT CD45.1^+^ mice (5 x 10^6^ cells/mouse). 8 weeks later, peripheral blood of recipients was tested. **(A)** Flow cytometry of B220^+^ B cells, CD4^+^ T cells and CD8^+^ T cells in chimera mice at day60 post cell transfer. **(B)** Flow cytometry of WT (CD45.1^+^) and Tbx21^-/-^ cells (CD45.2^+^) in B220^+^ B cells, CD4^+^ T cells and CD8^+^ T cells of chimera mice. Numbers adjacent to outlined areas in **(A,B)** indicate percent of each cell subset in parent subset.


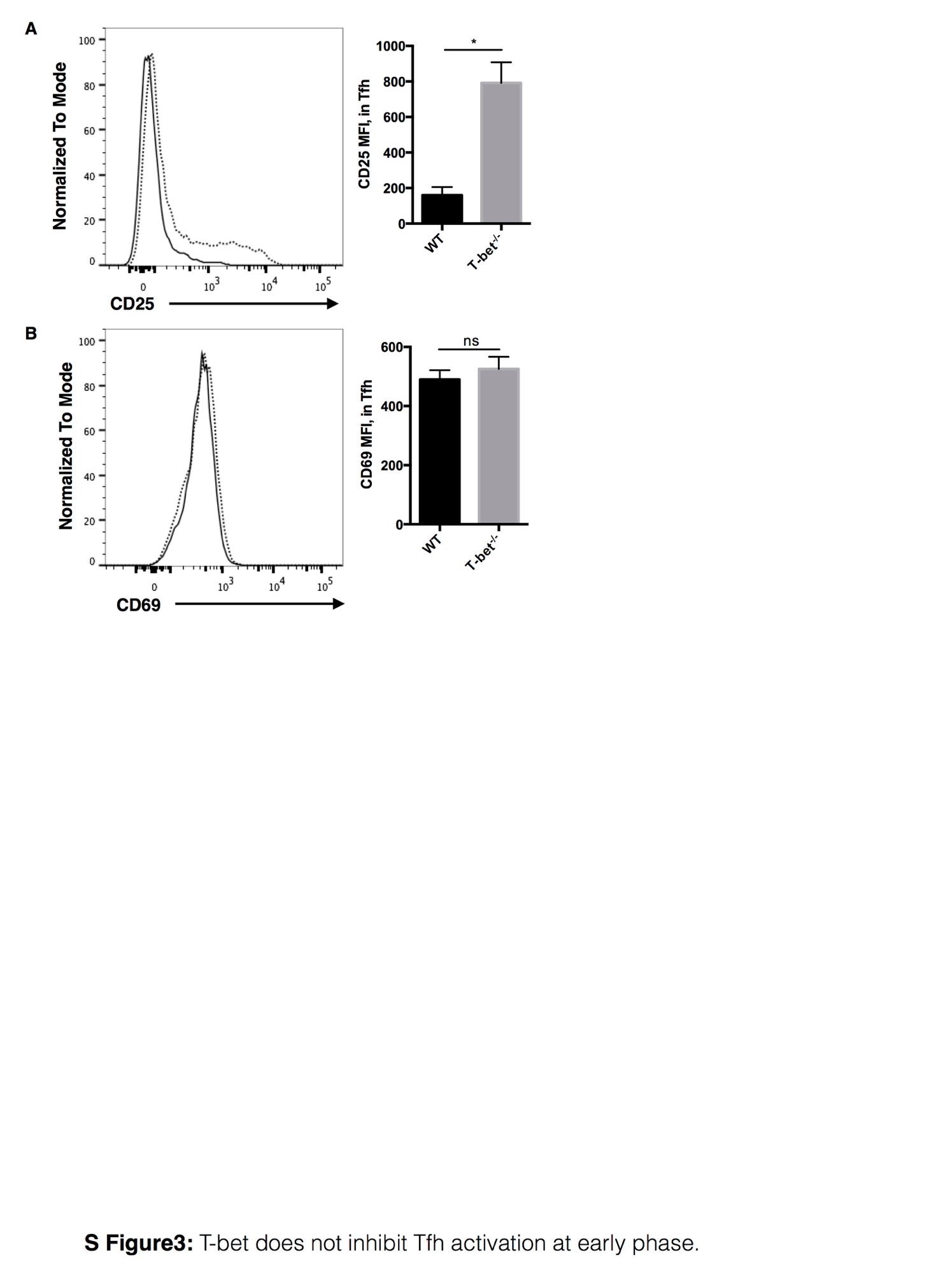


**S FIGURE 4 | T-bet does not inhibit Tfh activation at early phase (associated with Figure 6). (A)** Flow cytometry of CD25 expression, with the summary of CD25 expression (showed as MFI) in SMARTA TFH cells at day2 post infection. **(B)** Flow cytometry of CD69 expression, with the summary of CD69 expression (showed as MFI) in SMARTA TFH cells at day2 post infection. ns, not significant; **P* < 0.05 (unpaired two-tailed *t*-test). Data are representative of two independent experiments with 3-5 mice per group (error bars, SEM).


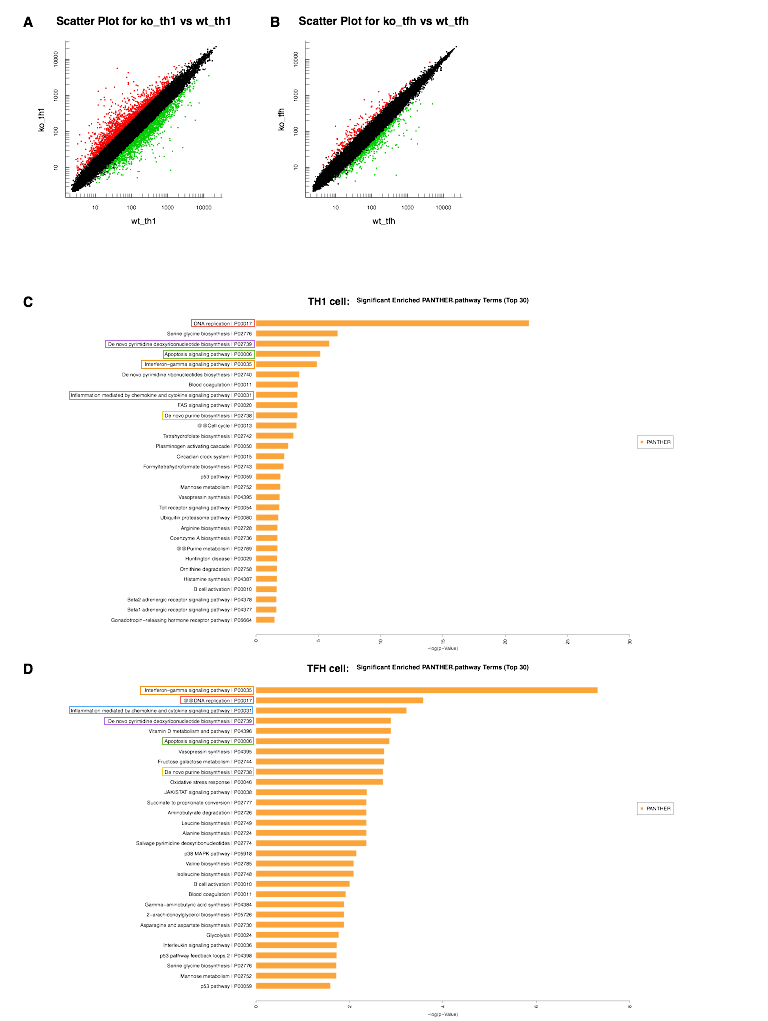


**S FIGURE 5 | Scatter plot of differentially expressed genes and Pathway enrichment of the TFH and TH1 cell transcriptomes (****associated with Figure 7). (A,B)** Scatter plot showing the differentially expressed genes in TH1 **(A)** and TFH **(B)** cells of Tbx21^-/-^ mice relative to WT mice. The red plots represent upregulated genes, the green plots represent downregulated genes, and the black plots represent genes without clear differences in expression between Tbx21^-/-^ and WT cells (fold change≥ 2). **(C)** PANTHER pathway enrichment analysis of TH1 cells (WT TH1*vs*Tbx21^-/-^ TH1 cells). **(D)** PANTHER pathway enrichment analysis of TFH cells (WT TFH*vs*Tbx21^-/-^ TFH cells).
